# Is intra-abdominal hypertension a missing factor that drives multiple organ dysfunction syndrome?

**DOI:** 10.1186/cc13785

**Published:** 2014-03-19

**Authors:** Andrew W Kirkpatrick, Derek J Roberts, Jan De Waele, Kevin Laupland

**Affiliations:** 1 Department of Surgery, University of Calgary, Foothills Medical Centre, Calgary, Alberta T2N 2T9, Canada; 2 Department of Community Health Sciences, University of Calgary, Foothills Medical Centre, Calgary, Alberta T2N 2T9, Canada; 3 Regional Trauma Program, University of Calgary, Foothills Medical Centre, Calgary, Alberta T2N 2T9, Canada; 4 Department of Critical Care Medicine, Ghent University Hospital, 9000 Ghent, Belgium; 5 Department of Medicine, Royal Inland Hospital, Kamloops, British Columbia V2C 2T1, Canada

## Abstract

In a recent issue of *Critical Care*, Cheng and colleagues conducted a rabbit model study that demonstrated that intra-abdominal hypertension (IAH) may damage both gut anatomy and function. With only 6 hours of IAH at 25 mmHg, these authors observed an 80% reduction in mucosal blood flow, an exponential increase in mucosal permeability, and erosion and necrosis of the jejunal villi. Such dramatic findings should remind all caring for the critically ill that IAH may severely damage the normal gut barrier functions and thus may be reasonably expected to facilitate bacterial and mediator translocation. The potential contribution of IAH as a confounding factor in the efficacy of selective decontamination of the digestive tract should be considered.

## 

Using a rabbit model, Cheng and colleagues [1] demonstrated that intra-abdominal hypertension (IAH) dramatically damages the anatomy and function of the gastrointestinal tract (gut). The authors utilized a rigorous design to examine gut form and function subjected to IAH through multiple methodologies, namely by studying perfusion, intestinal permeability, and the structural integrity by both light and electron microscopy [1].

The gut represents the largest body surface in contact with the external environment and constitutes a reservoir of more than 100 trillion bacteria [2]. However, unlike with many other organs and/or body systems that are readily monitored clinically, radiologically, or through invasive techniques, clinical examination of the abdomen represents the standard for gut function monitoring. The gut therefore may be considered a clinically silent organ system that may be a ‘ticking time-bomb’ in many admitted to the critical care unit. Multiple organ dysfunction syndrome (MODS) is well feared as a leading cause of morbidity, mortality, and resource use in critical care units [3,4]. For more than half a century it has been hypothesized that the gut is the motor of a catastrophic sequence of events that ultimately results in MODS [2,5]. The theory is that translocation of aerobic Gram-negative bacilli and/or absorption of their resultant endotoxin contributes to a systemic inflammatory state resulting in widespread organ dysfunction, tissue damage, and subsequent MODS [5].

Postulation of the gut in this role has led to the development of selective digestive decontamination (SDD) protocols wherein antimicrobials are utilized to reduce gut bacterial load. These protocols utilize both broad-spectrum parenteral and enteral antimicrobials, and have been demonstrated to improve outcome related to a reduction of the severity and/or incidence of MODS by the prevention of infection, gut overgrowth, aerobic Gram-negative bacilli translocation, and endotoxin absorption [5,6]. This seemingly simple solution to such a challenging problem has tantalized generations of physicians. Gut decontamination is one of the most persistently researched areas in critical care with no less than 65 randomized trials involving over 15,000 patients that have generated 11 meta-analyses by the end of 2011 [7]. Despite such efforts, however, use of SDD remains highly controversial, with widely divergent opinions ranging from scepticism [7,8] to belief that it represents standard of care [7,9]. Some of these divergent views arise related to concerns about development of anti-microbial resistance as well as from the analysis of the data involving subgroup analysis, surrogate outcomes, and *post hoc* analyses [5]. Continuing investigations are thus underway in an effort to better understand how SDD may work, who it may best benefit, and how it may influence emergence of resistant organisms.

The current study by Cheng and associates [1] raises a number of important questions surrounding the genesis of MODS and the use of SDD. It is commonly assumed that shock is the precipitating factor that results in gut hypoperfusion and initiation of MODS, and this seems likely to be the case in many patients who present with early shock. However, it poorly explains the later development of MODS in patients where shock resuscitation has been prompt. There is an increasing body of evidence that development of IAH may be a major contributor in this case. The theory and rationale underlying the potential benefit of SDD on the other hand is related to reducing organism burden and subsequent translocation by a sickened gut. As an upstream event, enhanced measures to maintain gut health, such as preventing IAH, might be of greater value in preventing MODS. At the least, future SDD investigations need to consider and control for IAH as a potential covariate.

The results of Cheng and associates are important in emphasizing the dangers of IAH. The World Society of the Abdominal Compartment Syndrome recently published updated Consensus Guidelines [10], which revalidated the definition and gradation of IAH as originally proposed to understand and study concerns regarding pathologically raised intra-abdominal pressure in the critically ill [11,12]. IAH is classified as grade I, 12 to 15 mmHg; grade II, 15 to 20 mmHg; grade III, 20 to 25 mmHg; and grade IV, >25 mmHg [10]. While it is uncertain and even unlikely that pressure thresholds derived from animals completely correlate with human values, it is pertinent that what might erroneously be considered ‘mild’ grade I IAH (15 mmHg) had profound effects on mucosal blood flow, which was reduced by 50% after only 4 hours. When IAH was even more severe (in the range commensurate with abdominal compartment syndrome (defined as intra-abdominal pressure >20 associated with new organ failure [10,13])), there was profound injury to the gut. Nearly 20 years ago Diebel and colleagues [14] demonstrated that similar degrees of IAH in a rodent model were associated with bacterial translocation to the mesenteric lymph nodes. To date, however, no group has correlated the dose-effect of the pathophysiology and histology of IAH-induced gut injury so elegantly as Cheng and colleagues. Generalizing results of animal model studies to bedside management of patients must be done with caution, as it is recognized that such studies will never be truly replicated clinically. In any case, this important study conducted by Cheng and colleagues should be a prompt for greater recognition of the adverse effects of IAH in the critically ill and stresses the importance of evaluating IAH as a covariate in future studies investigating the pathophysiology or management of MODS.

## Abbreviations

IAH: Intra-abdominal hypertension; MODS: Multiple organ dysfunction syndrome; SDD: Selective digestive decontamination.

## Competing interests

AWK and DJR are participating in a randomized trial of open abdomen management with unrestricted funding from the KCI Corporation. AWK has received compensation to attend cadaver labs from the Synthes and LifeCell Corporations. JDeW and AWK are President and President Elect of the World Society of the Abdominal Compartment Syndrome. DJR is funded by the Alberta Innovates - Health Solutions Clinician Fellowship award. KL has no competing interest to declare.
